# Probiotic-based strategies for therapeutic and prophylactic use against multiple gastrointestinal diseases

**DOI:** 10.3389/fmicb.2015.00685

**Published:** 2015-07-14

**Authors:** Natallia V. Varankovich, Michael T. Nickerson, Darren R. Korber

**Affiliations:** Department of Food and Bioproduct Sciences, University of Saskatchewan, SaskatoonSK, Canada

**Keywords:** probiotic, human gut, gastrointestinal disorders, antibiotic resistance, gut microbiota

## Abstract

Probiotic bacteria offer a number of potential health benefits when administered in sufficient amounts that in part include reducing the number of harmful organisms in the intestine, producing antimicrobial substances and stimulating the body’s immune response. However, precisely elucidating the probiotic effect of a specific bacterium has been challenging due to the complexity of the gut’s microbial ecosystem and a lack of definitive means for its characterization. This review provides an overview of widely used and recently described probiotics, their impact on the human’s gut microflora as a preventative treatment of disease, human/animal models being used to help show efficacy, and discusses the potential use of probiotics in gastrointestinal diseases associated with antibiotic administration.

## Microbial Ecology of the Human Gastrointestinal Tract

The human intestinal microbiota is a complex ecosystem with considerable impact on human health and well-being, contributing to maturation of the immune system and providing a direct barrier against pathogen colonization (Doré and Corthier, 2010). It consists of bacteria, archaea, some protozoa, anaerobic fungi, and different bacteriophages and viruses, and it has been estimated that more than 1000 species of microbes inhabit the human intestine (Tuohy et al., 2012). The presence of a great number of microbes (up to 5 × 10^11^ bacterial cells per gram of intestinal contents) suggests strong regulatory effects on the human host, and recent findings suggest that gut microbiota can have a considerable impact on both our weight and mood (Duca et al., 2014; Naseribafrouei et al., 2014). The composition and function of human microbial populations associated with various body sites have been studied with the help of metagenomic tools as part of two recent initiatives – the NIH Human Microbiome Project (HMP) and the European Metagenomics of the Human Intestine (metaHIT) project (NIH HMP Working Group et al., 2009; Dusko Ehrlich and MetaHIT Consortium, 2011). These massive molecular approaches have already revealed the presence of three different clusters, or enterotypes, which correspond to one of three most abundant genera of human intestine – *Bacteroides, Prevotella*, and *Ruminococcus* (Arumugam et al., 2013).

Bacteria that initially colonize the large gut of an infant are facultative anaerobes, such as *Escherichia coli* and *Streptococcus* sp. These species metabolize oxygen in the gut, thereby creating anaerobic conditions. Subsequent colonization largely depends on food profile and environmental factors (i.e., sanitary conditions). After the full formation of the gastrointestinal microflora, its composition has been shown to include such genera as *Bacteroides, Bifidobacterium, Eubacterium, Clostridium, Lactobacillus, Fusobacterium*, and various Gram-positive cocci (Fooks et al., 1999; Wallace et al., 2011).

Within the gastrointestinal tract (GIT), the microbiota provides various functions, such as digestion of essential nutrients and maturation of intestinal epithelial cells. Studies on mice have shown a number of significant effects of microbiota on the host: in ex-germ-free reconventionalized mice, their intestinal epithelium was thicker, kinetics of enterocytes – faster, short-chain fatty acids were produced at significantly higher concentrations, and there was a normal level of immunological activity present, compared to germ-free animals (Aureli et al., 2011). Microbes also have the ability to affect physiologic parameters, providing systemic effects on blood lipids and generally influencing the immune system, as well as inhibiting harmful bacteria (Mikelsaar, 2011). Pathogen inhibition by human intestinal microbiota may provide significant human health benefits through protection against infection as a natural barrier against pathogen exposure in the GIT (Wallace et al., 2011). Factors such as food contamination by pathogens, as well as the high load of antibiotics in soil and animal feed, can influence the microbial ecology of human GIT (Sapkota et al., 2007). Using molecular genetic tools, it has been shown that antibiotics could induce significant alterations in the dominant colonic microbiota that are not detectable using bacteriological (culture-based) techniques, with effects lasting for up to 2 months (Mangin et al., 1999). Several more specific disorders involve disruption of the human microflora ecology: acute gastroenteritis, *Clostridium difficile* infection (CDI), necrotising enterocolitis in neonates, irritable bowel syndrome and *Helicobacter pylori* infection (Kotzampassi and Giamarellos-Bourboulis, 2012). Probiotics are currently being examined for their potential treatments of these aforementioned disorders.

## Probiotic Bacteria

According to the popularized definition by the Food and Agriculture Organization/World Health Organization, and as grammatically modified by Hill et al. (2014), probiotics are defined as “Live microorganisms that, when administered in adequate amounts, confer a health benefit on the host” (FAO/WHO, 2001). The most common probiotics include representatives of lactobacilli, enterococci, bifidobacteria, and yeasts (**Table 1**). In addition, bacterial mixtures may be used to achieve the complex beneficial effect of probiotics (Caballero-Franco et al., 2007).

**Table 1:** Microorganisms with reported probiotic potential.

Probiotic group	Species	Details of a representative study	Reference
Bifidobacteria	*Bifidobacterium animalis*	Fermented oat milk with *B. animalis* sp. *lactis* BB-12^®^; double-blind randomized placebo-controlled clinical trial; *n* = 209; 35% improvement in bowel movement.	Pitkala et al. (2007), Jungersen et al. (2014)
	*B. breve*	*B. breve* M16-V powder with or without starch; F344/Du rat pups; *n* = 46; downregulation of the expression of inflammatory molecules.	Shimakawa et al. (2003)
	*B. adolescentis*	*B. adolescentis* ATCC 101; female germ-free rats; *n* = 30; significant modulation of both systemic and the intestinal immune response to *Bacteroides thetaiotaomicron* DSMZ 2079.	Scharek et al. (2000)
	*B. longum*	*B. longum*, isolated from human GIT; double-blind randomized placebo-controlled clinical trial; *n* = 29; significant inhibitory effect on viral gastroenteritis symptoms.	Lee et al. (2014b)
	*B. infantis*	*B. infantis* 35624 was administered by gavage; C57BL/6 mice; *n* = 64; decrease in the severity of dextran sulfate sodium-induced colitis, immunomodulation.	Konieczna et al. (2013)
Lactobacilli	*L. acidophilus*	*L. acidophilus –* SDC, administered in capsules; double-blind randomized placebo-controlled clinical trial; *n* = 40; 23.6% reduction (compared to placebo) in the severity (pain, discomfort) of Irritable Bowel Syndrome.	Sinn et al. (2008)
	*L. fermentum, L. amylovorus*	Microencapsulated bacteria; double-blind randomized placebo controlled clinical trial; *n* = 28, obese adults; *L. fermentum –* 3% loss in total fat mass, *L. amylovorus –* 4% loss in total fat mass, a significant reduction in the abundance of Clostridial cluster IV.	Omar et al. (2013)
	*L. rhamnosus*	*L. rhamnosus GG* powder; double-blind randomized clinical trial; n = 559; decrease in frequency and duration of acute watery diarrhea.	Basu et al. (2009)
	*L. paracasei*	*L. paracasei* ST11, lyophilized form; double-blind randomized placebo-controlled clinical trial; *n* = 230, male infants and young children; significant benefit in the management of children with non-rotavirus-induced diarrhea.	Sarker et al. (2005)
	*L. johnsonii*	*L. johnsonii* La1 in dietary product; double-blind randomized placebo-controlled clinical trial; *n* = 326, children, found positive for *Helicobacter pylori*; significant decrease in σ^13^CO_2_ above baseline values (outcome of a test for *H. pylori*).	Cruchet et al. (2003)
	*L. reuteri*	*L. reuteri* SD 2112; randomized controlled clinical tral; *n* = 40, infants and children; significant decrease in diarrhea symptoms.	Shornikova et al. (1997)
	*Pediococcus pentosaceus*	*P. pentosaceus* NB-17; mouse spleen cells were co-cultivated with heat-killed bacteria; *in vitro* investigation of the production of cytokines; effective stimulation of immune activities and allergic inhibitory effects.	Jonganurakkun et al. (2008)
	*Oenococcus oeni*	*O. oeni* 9115; female BALB/c mice with 2, 4, 6-trinitrobenzene sulfonic acid-induced experimental colitis; *n* = 20; significant decrease in severity of colitis. Several *O. oeni* strains were able to modulate the immune response of immunocompetent cells *in vitro.*	Foligné et al. (2010)
Enterococci	*E. durans*	*E. durans* LAB18s; *in vitro* study; antimicrobial activity, antioxidant ability, evidenced in both culture supernatants and intracellular extracts; resistance to acidic conditions (pH 3) and bile salts.	Pieniz et al. (2014)
	*E. faecium*	*E. faecium* MMRA; *in vitro* study; genes, coding enterocins A, B, P and X; high survival rates under stress caused by acidic pHs (2-5) or bile salts (0.3%), and a high adhesive potential.	Rehaiem et al. (2014)
	*E. faecalis*	*E. faecalis* UGRA10; *in vitro* study; production of AS-48 enterocin; ability to form biofilms and to adhere to Caco 2 and HeLa 229 cells.	Cebrián et al. (2012)
	*E. lactis*	*E. lactis* IITRHR1 was administered by gavage; male Wistar rats with acetaminophen-induced hepatotoxicity; *n* = 42; pretreatment with the bacterium lowered the level of biomarkers of hepatotoxicity in serum; significant increase in the level of antioxidants; modulation of key apoptotic/anti-apoptotic proteins (cytochrome-c, Bcl2, Bax, expression of caspases).	Sharma et al. (2012)
Yeasts	*Saccharomyces boulardii*	Granulated *S. boulardii*; double-blind randomized placebo-controlled clinical trial; *n* = 200, children with acute diarrhea; significant decrease in severity of symptoms and duration of hospital stay.	Kurugol and Koturoglu (2005), Kelesidis and Pothoulakis (2012)

Presumed health benefits of probiotics include reducing harmful organisms in the intestine, producing antimicrobial factors, and stimulating the body’s immune response (Collado et al., 2007; Foligné et al., 2010; Konieczna et al., 2013). Some of the beneficial effects of probiotics (e.g., lowering of cholesterol level) are yet to be substantiated by well-controlled clinical trials. However, there are a growing number of studies providing data on effects of probiotic bacteria on the human immune system and on microflora of the GIT (Holzapfel and Schillinger, 2002; Foligne et al., 2007; Verdú et al., 2009; Wen et al., 2012). Increasingly, reports of the human/animal microbiome playing a central role in other key aspects of health functionality are emerging, including beneficial impacts on the treatment of metabolic disorders, such as obesity and type 2 diabetes, improvement of bowel function in patients with colorectal cancer, potential cognitive, and mood-enhancing benefits, antidepressant, and anxiolytic (antianxiety) activity (Desbonnet et al., 2008; Bravo et al., 2011; DiBaise et al., 2012; Lee et al., 2014a; Owen et al., 2014). The latter anxiolytic effect has even led to the emergence of the new term, psychobiotic, coined by Dinan et al. (2013) as a “live organism that, when ingested in adequate amounts, produces a health benefit in patients suffering from psychiatric illness.”

Products containing probiotic bacteria generally include supplements and foods. Live probiotics are commonly available in fermented dairy products and probiotic-fortified foods. These bacteria are added into numerous foods and beverages, ranging from yogurts to breakfast cereals. There are also tablets, capsules, powders, and sachets containing probiotics in freeze-dried form. Functional foods, defined as food preparations with various health-related properties, often include bacterial strains with declared probiotic properties (Turroni et al., 2011). The scientific interest in probiotics is growing exponentially: the search for published papers featuring the keyword “probiotic” in NIH PubMed database revealed 7265 articles for the period from 2000 to 2010, with 953 of them being clinical trials. Within the following 5 years (up to May 20th 2015), the frequency of publications doubled with 7979 papers being published, including 778 clinical trials.

### Lactic Acid Bacteria

Lactic acid bacteria (LAB) are Gram-positive, non-spore forming cocci, coccobacilli, or rods, which generally have non-respiratory (fermentative) metabolism and lack true catalase. Unlike bifidobacteria, which are active in lower parts of the colon, lactobacilli are prevalent in the upper GIT (Turroni et al., 2011). This group is also a normal member of the human microflora, found in the oral cavity, the small intestine, and the vaginal epithelium, where it is thought to play beneficial roles (Gomes and Malcata, 1999). Among the beneficial effects, lactobacilli can improve digestion, absorption, and availability of nutrients (Wallace et al., 2011). Furthermore, LAB are capable of hydrolyzing compounds that limit the bioavailability of minerals, like tannin and phytate, due to tannin acylhydrolase and phytase activities (Turpin et al., 2010). In addition, it was shown that some lactobacilli strains could enhance mineral absorption in Caco-2 cells and improve the nutritional status of the host by producing B-group vitamins. More recently, the role of lactobacilli in energy homeostasis, particularly in obese patients, has been the object of an increased interest (Guo et al., 2010; Mikelsaar, 2011). A further potential positive impact of LAB is their ability to inhibit or kill *H. pylori*, which is now regarded as the major cause of gastritis and peptic ulcers and is a risk factor for gastric malignancy (Hamilton-Miller, 2003). In addition, both *Lactobacillus* sp. and *Bifidobacterium* sp. strains can reduce the side effects of *H. pylori* eradication therapy (Canducci et al., 2002).

Pediococci are also related to the LAB group and are utilized in industrial fermentations of foods and silage (Raccach, 2014). Pediocin-producing *Pediococcus* sp. strains are of potential interest to food safety (Raccach, 2014), with three of them potentially possessing probiotic properties – *Pediococcus pentosaceus, P. parvulus*, and *P. acidilactici*. Osmanagaoglu et al. (2010) comprehensively studied the potential of a human *P. pentosaceus* isolate for probiotic use, and reported that the strain produced an anti-*Listerial* bacteriocin, had excellent autoaggregation characteristics and was also able to co-aggregate with *Salmonella enterica* serotype typhimurium and enterotoxigenic *Escherichia coli* (Osmanagaoglu et al., 2010). Antagonistic activity against *Listeria monocytogenes* was also discovered in *P. acidilactici* (Guerra and Pastrana, 2002). Clinical trials employing another strain of *Pediococcus* sp. revealed that the administration of *P. parvulus* decreased the serum cholesterol levels and increased counts of fecal *Bifidobacterium* sp. (Mårtensson et al., 2005).

Another group of LAB promoted as probiotics are enterococci, which reportedly help in the maintenance of normal intestinal microflora and stimulate the immune system (Bhardwaj et al., 2008). Studies of potential probiotic properties of *E. faecium* showed its efficacy in reducing the recovery period of acute diarrhea (Benyacoub et al., 2003). Another study by Pieniz et al. (2014) showed that *E. durans* possessed antimicrobial activity and antioxidant ability and was resistant to simulated gastric juice and bile salts. Though enterococci have probiotic potential, they are considered opportunistic pathogens for humans as they might cause nosocomial infection and are also known to possess resistance to vancomycin (Tambyah et al., 2004). Due to these controversial properties, the use of enterococci as probiotics remains under debate.

### Bifidobacteria

Bifidobacteria are major constituents of the GIT microbiota of animals and humans. They are Gram-positive, non-motile anaerobic saccharolytic bacteria (Gomes and Malcata, 1999). In the gut environment, bifidobacteria have a commensal relationship with their hosts, and contribute to host nutrition by utilizing complex carbohydrates, which are important sources of carbon and energy, but are not degraded in the stomach or intestine (Biavati, 1994). These substances include plant-derived dietary fiber and diet-related carbohydrates, such as starch, galactan, sucrose, amylopectin, and pullulan (Ventura et al., 2007, 2012). The capacity of bifidobacteria to metabolize non-digestible host dietary carbohydrates (prebiotics) can be used for selective stimulation of certain strains colonizing the intestinal tract. Bifidobacteria used as probiotics include strains belonging to species of *Bifidobacterium lactis, B. bifidum, B. animalis, B. thermophilum, B. breve, B. longum, B. infantis*, and *B. adolescentis* (**Table 1**). These bacteria have been shown to inhibit the adherence of enterotoxigenic *E. coli*, enteropathogenic *E. coli*, and *C. difficile* to intestinal epithelial cells, an important trait for use of these bacteria as probiotics (Tsai et al., 2008). Additional beneficial effects of bifidobacterial strains include the prevention or alleviation of infectious diarrhea and the improvement of inflammatory bowel disease symptoms (Sanz, 2007). Bifidobacteria have also been shown to modulate the host’s immune response against other indigenous microflora (e.g., *B. adolescentis* down-regulates humoral immunity to *Bacteroides thetaiotaomicron*; Scharek et al., 2000). Some bifidobacterial strains suppress *H. pylori*-induced genes in human epithelial cells (Shirasawa et al., 2010) while other *Bifidobacterium* sp. cells and culture supernatants exerted inhibitory effects against *Streptococcus mutans* and *Streptococcus sobrinus*, important etiological agents in human dental caries (Lee et al., 2011).

### Yeasts

*Saccharomyces boulardii* is one of the best-studied probiotic species, with a long history of successful use in treatment of multiple gastrointestinal disorders. The administration of this probiotic in lyophilized form was found effective in cases of diarrhea by decreasing the duration of the disease, regardless of its cause (McFarland, 2007; Dinleyici et al., 2012; Shan et al., 2013). It has also been reported that *S. boulardii* prevented and treated relapses of inflammatory bowel disease, including moderate cases of ulcerative colitis (Guslandi et al., 2000; Guslandi et al., 2003; Choi et al., 2011). Interesting results have also been reported by Lim et al. (2015), suggesting that yeasts can enhance the growth of other probiotics under acidic conditions: *Saccharomyces cerevisiae* EC-1118 was found to significantly enhance the viability of the probiotic strain *Lactobacillus rhamnosus* HN001 at pH 2.5–4.0. The use of *S. boulardii* in reduction of *C. difficile* infection relapse is still under debate due to controversial results of clinical trials (Flatley et al., 2015). Among other yeasts species, *Torulaspora delbrueckii, Debaromyces hansenii, Yarrowia lipolytica, Kluyveromyces lactis, Kluyveromyces marxianus, and Kluyveromyces lodderae* have shown strong antagonistic effect against pathogenic bacteria and high acid tolerance (Kumura et al., 2004; Psani and Kotzekidou, 2006; Chen et al., 2010). Despite an excellent record of safe use, yeasts may still be the cause of localized infections in immunocompromised patients (Thygesen et al., 2012).

### Akkermansia muciniphila

Another recently described microorganism with possible probiotic potential is *Akkermansia muciniphila* – a mucin-degrading bacterium that resides within intestinal mucus layers (Derrien et al., 2004). According to several studies, obese patients have significantly lower amounts of this bacterium in their GIT (Collado et al., 2008; Karlsson et al., 2012). The genome sequence of *A. muciniphila* suggests the ability of this bacterium to metabolize a variety of complex carbohydrates, as well as synthesize multiple amino acids, vitamins, and cofactors (van Passel et al., 2011). Its influence on metabolic processes in the GIT is not fully investigated; however, it has already been shown that this bacterium may be a potential treatment for type II diabetes. Shin et al. (2014) have shown that oral administration of *A. muciniphila* to mice induced Foxp3 regulatory T cells in the visceral adipose tissue, which attenuated adipose tissue inflammation. Based on these results it has been suggested that pharmacological manipulation of the gut microbiota in favor of *A. muciniphila* might be beneficial in the treatment of diabetes.

### *Faecalibacterium prausnitzii* and Other Clostridia

Another bacterium that has been demonstrated to have a considerable impact on human gastrointestinal microbiota is *Faecalibacterium prausnitzii* of the *Clostridium* sp. cluster IV. This microorganism accounts for 5–15% of the total fecal microbiota, making it one of the most abundant butyrate-producing bacteria in the GIT (Hold et al., 2003; Flint et al., 2012). Since butyrate is a primary energy source for intestinal epithelial cells, it is essential for maintenance of epithelial barrier integrity. Multiple beneficial effects of butyrate for health also include reduction of cancer progression, protection against pathogens, and stimulation of the immune system (Macfarlane and Macfarlane, 2011). The reduction of *F. prausnitzii* counts in fecal and biopsy samples has been observed in multiple studies of inflammatory bowel disease (especially, ileal Crohn’s disease and ulcerative colitis), suggesting that the presence of this species is important for normal GIT function (Wang et al., 2007; Swidsinski et al., 2008; Andoh et al., 2012). The first gnotobiotic rodent model with *F. prausnitzii* showed that it could influence gut physiology through the production of mucus *O*-glycans, thereby affecting the quality and quantity of produced mucus (Wrzosek et al., 2013). Though *F. prausnitzii* dysbiosis might be an important marker in the development of disease, routine diagnostic tools have not been developed mainly due to the extreme sensitivity of this species to oxygen.

Other bacteria of the class Clostridia might also find use as potential probiotics, since they are highly abundant in human GIT microbiota and may play an important role in metabolism and immune system function. Atarashi et al. (2013) have shown that a mixture of 17 strains of *Clostridium* sp., belonging to clusters IV, XIV, and XVIII, were able to suppress experimental colitis in mice through induction of interleukin-10-producing regulatory T cells. A similar mechanism of colitis suppression, via IL-10 production by induced macrophages, was observed using strain *C. butyricum* MIYAIRI 588 (Hayashi et al., 2013). According to another recent study, when mixed with *B. infantis, C. butyricum* was effective in treatment of experimentally-induced antibiotic-associated diarrhea in mice, and the beneficial effect of the mixture was superior to single strains (Ling et al., 2015). However, though clostridia have potential for use as probiotics, there is still not enough evidence to support their medical efficacy and safety for humans.

## Use of Probiotics in Prevention and Treatment of Antibiotic-Associated Diseases

Although most antibiotics are generally safe, some have the potential to cause life-threatening side effects. Antimicrobial side effects are adverse drug reactions involving one or more organ systems. Moreover, even a short-term course of antibiotics may have a long-term negative impact on the normal human gut microbiota (Jernberg et al., 2010). The most commonly used classes of antibiotics include penicillins, cephalosporins, aminoglycosides, fluoroquinolones, macrolides, and tetracyclines; each of these compounds can cause their own specific side-effects (Cunha, 2001). In fact, most traditionally used antibiotics are able to cause health problems in the GIT, and are commonly related to disturbances in microflora composition caused by survival and spread of resistant strains. For instance, penicillins, which are known for having the least-frequent and -severe side effects, may cause diarrhea, and nausea, vomiting, and upset stomach. Fluoroquinolones are also considered relatively safe, but may similarly induce nausea, vomiting, diarrhea, and abdominal pain (Bertino and Fish, 2000). Side-effects of macrolides include GIT-associated nausea, vomiting, and diarrhea, whereas adverse effects of the tetracyclines depend on the concentration of the antibiotic in the affected organ. Their common side-effects include cramps or burning of the stomach, diarrhea, sore mouth, or tongue (Rubinstein, 2001). Research in this field is ongoing and has already provided evidence for efficacy of probiotic use for prevention of health problems emerging as a result of antibiotic use. Examples of such diseases are antibiotic-associated diarrhea (AAD) and *C. difficile*-associated diarrhea (CDAD; pseudomembranous colitis).

Antibiotic-associated diarrhea is defined as “otherwise unexplained diarrhea that occurs in association with the administration of antibiotics” (Friedman, 2012). However, mild cases of *C. difficile* infection are sometimes also considered as the cause of AAD (Kelly et al., 1994). The disease comes as one of the most frequent side effects of antibiotic use: 5–39% of patients, depending on the type of antibiotic (e.g., certain β-lactam antibiotics are more likely to cause diarrheal side-effects than cephalosporins) and is associated with increased length and cost of hospitalization (Videlock and Cremonini, 2012). There are several mechanisms of antibiotic effect on humans that can result in AAD. These include osmotic diarrhea, caused by suppression of anaerobic bacteria and a reduction in carbohydrate metabolism, disruption of protective effect of commensal bacteria and reduction of colonic mucosal resistance to pathogenic opportunistic bacteria. Full restoration of the normal gut microbiota may take several weeks or even months (Friedman, 2012; Kaier, 2012).

Many studies have been conducted assessing the efficacy of probiotics in the treatment of AAD and have provided data supporting the usage of both single-strain and mixed-probiotics for diarrhea treatment (Surawicz, 2003; Szajewska et al., 2006; McFarland, 2009). A meta-analysis by Hempel et al. (2012) revealed 82 studies that provided evidence of probiotic efficiency in treatment of AAD. Microorganisms used in these studies included the genera *Lactobacillus, Bifidobacterium, Saccharomyces, Streptococcus, Enterococcus*, and *Bacillus.* According to Friedman (2012), several mechanisms of action of probiotics contribute to the prevention and treatment of diarrhea: enhancing mucosal barrier function by secreting mucins, increasing tight junctions in epithelial cells, providing colonization resistance, producing bacteriocins, increasing production of secretory lgA, producing balanced T-helper cell response, increasing production of IL-10 and transforming growth factor beta. Collectively, these factors contribute to the restoration of a normal gastrointestinal balance following damage by antibiotics (Friedman, 2012).

*Clostridium difficile*-associated diarrhea or pseudomembranous colitisis is an inflammation of the intestine walls caused by toxins produced by *C. difficile*. CDAD is one of the most common hospital-acquired infections and is a frequent cause of morbidity and mortality among elderly hospitalized patients. Complications include shock, need for colectomy, toxic megacolon, and in severe cases, perforation of the colon wall. *C. difficile* colonizes the GIT after the alteration of normal gut flora by antibiotic therapy (Bergogne-Bérézin, 2000; Ndegwa and Nkansah, 2008). Extremely high rates of CDAD have been reported in Quebec from 2002 to 2005, totaling 14000 cases (a 4.5-fold increased incidence compared with 1991), with evidence suggesting the emergence of a highly-virulent strain of *C. difficile* (Pepin et al., 2004, 2005).

Several studies have shown that probiotics aid in prevention and treatment of CDAD. Gao et al. (2010) reported lower risk of disease occurrence after intake of a preparation based on two *Lactobacillus* strains. *S. boulardii* has also been successfully used for treatment of CDAD (McFarland et al., 1994). However, a large multi-center study is needed to build sufficient evidence in support of probiotic use as a treatment for *C. difficile-*associated infections.

### Problems Associated with Transfer of Antibiotic Resistance Determinants

Many probiotic strains have naturally acquired resistance toward one or several antimicrobial agents (**Table 2**). Though intrinsic resistance of probiotic bacteria to certain antibiotics might offer benefits for their use in the prevention and treatment of AAD, the issue of possible transfer of resistance determinants has been raised (Pflughoeft and Versalovic, 2012), particularly for strains that carry plasmids.

**Table 2:** Intrinsic antibiotic resistance of several widely used probiotic species.

Probiotic species	Antibiotic resistance	Reference
*B. longum JDM301*	Ciprofloxacin, amikacin, gentamicin, streptomycin	Wei et al. (2012)
*B. longum^2^*	Kanamycin	Temmerman et al. (2003)
*B. lactis^2^*	Kanamycin	Temmerman et al. (2003)
*L. rhamnosus^1^*	Vancomycin, teicoplanin, bacitracin, aminoglycosides	Charteris et al. (1998), Danielsen and Wind (2003)
*L. casei^1^*		
*L. paracasei^1^*		
*L. plantarum^1^*		
*L. acidophilus^1^*		
*L. reuteri^2^*	Kanamycin, tetracycin, penicillin, vancomycin	Temmerman et al. (2003)
*L. casei^2^*	Kanamycin, vancomycin	Temmerman et al. (2003)
*L. acidophilus^2^*	Kanamycin	Temmerman et al. (2003)
*E. faecalis^1^*	Beta-lactams	Yamaguchi et al. (2013)
	Vancomycin	Werner et al. (2008)
*B. adolescentis^2^*	Kanamycin, neomycin, streptomycin, nalidixic acid	Kheadr et al. (2004)
*B. animalis^2^*		
*B. longum^2^*		
*B. bifidum^2^*		
*P. pentosaceus*	Vancomycin, teicoplanin	Biavasco et al. (1997)
*P. acidilactici*	Vancomycin	Temmerman et al. (2003)

*^1^Antibiotic resistance is indicated for more than 50% of isolates/strains used in the study, which were related to the species.*

*^2^Antibiotic resistance is indicated for 100% isolates/strains used in the study, which were related to the species.*

Courvalin (2006) specified two distinct types of acquired antibiotic resistance in bacteria: (i) initially non-transferred resistance that occurred as a result of one or several mutations in indigenous gene(s), and (ii) transferred resistance, acquired from a different organism by horizontal gene transfer. Antibiotic resistance (both intrinsic and acquired) can occur as a result of three major mechanisms: (i) altering the outer- and/or inner membrane permeability and transport activity, which leads to lower accumulation of the antibiotic within the cell, (ii) using enzymes to detoxify the antibiotic, and (iii) modifying the antibiotic target site (Guardabassi and Courvalin, 2006). The gene responsible for acquisition of antibiotic resistance often resides on a plasmid or transposon, which might be easily transferred (Bennett, 2008). In fact, transposon-mediated transfer of genetic material between species was recently described as the most frequent mechanism contributing to the spread of antibiotic resistance in bacteria (Wozniak and Waldor, 2010).

Multiple studies have already shown that antibiotic resistance can be transferred between different bacterial species that reside in the human GIT. For instance, it has been reported that both *Lactobacillus lactis* and *Streptococcus thermophilus* are able to transfer erythromycin resistance [*erm(B)* gene, located on a plasmid] to *L. monocytogenes* under *in vitro* conditions (Toomey et al., 2009). Another study provided the evidence of *in vivo* transfer of ampicillin resistance between two strains of *E. coli* co-residing in human gut: it was demonstrated that a plasmid carrying a β-lactamase gene had been transferred from an ampicillin- resistant *E. coli* strain to an initially susceptible strain (Karami et al., 2007). Devirgiliis et al. (2009) reported the transfer of a *tet*(M) gene (tetracycline resistance; located on broad host range Tn*916* transposon) from *L. paracasei* to *E. faecalis in vitro*. In another set of experiments, the erythromycin resistance pLFE1 plasmid of *L. plantarum* strain M345 was successfully transferred to five different species: *L. rhamnosus, Lc. lactis, Listeria innocua, E. faecalis*, and *L. monocytogenes* (Feld et al., 2009). These and other examples raise a safety concern; strains to be used as probiotics should be carefully selected, and only those free of transferrable antibiotic-resistance determinants ought to be considered safe (Radulovic et al., 2012).

## *In Vitro* and *In Vivo* Systems Used to Study Probiotic Effects

Novel probiotic-based strategies for therapeutic and prophylactic use against multiple GIT diseases are gaining popularity worldwide. Their effectiveness has been predicted by numerous animal model studies and proven by extensive research involving humans. However, the initial step in confirming probiotic effects is the extensive characterization of a bacterial strain to be used as a probiotic, which is usually performed under *in vitro* conditions by studying bacterial acid resistance, bile resistance, carbon source utilization, and aggregative properties, or *ex vivo* for their ability to adhere to mammalian cells (Kotikalapudi et al., 2010; Wood et al., 2012). Similarly, probiotic delivery methods, such as lyophilization or encapsulation, are also tested for their protective potential *in vitro* under simulated gastric conditions (Klemmer et al., 2011; Wood et al., 2012; Khan et al., 2013; Wang et al., 2014). The most popular materials used for encapsulation of bacteria are alginate, carrageenans and gums, since they are easy to process, resistant to low pH and freezing, and are generally recognized as safe (Gbassi and Vandamme, 2012). We have recently reported the efficient delivery of *B. adolescentis*, encapsulated for this purpose in an alginate-pea protein protective matrix, into the lower gut of rats (Varankovich et al., 2015).

Apart from basic synthetic gastric juice solutions (low pH, 37°C), more complex systems have been developed, such as SHIME (Simulator of the Human Intestinal Microbial Ecosystem), designed to simulate different parts of the human GIT (Cook et al., 2012). Probiotic strains and methods for their delivery, preselected *in vitro*, are subsequently tested in animal models.

Traditionally used animal models include mice and rats. Larger animals like rabbits, dogs, and pigs are generally considered to have more common features with the physiology and microflora of the human GIT (Kararli, 1995). However, rodents are cheap, standardized, and have short life-cycles; thus, their extensive use in large-scale research. Investigation of probiotic effects on animal microflora may be approached by: (i) examining the quantitative and qualitative characteristics of bacterial microflora in animals using cultivation and/or molecular biology techniques, such as real-time polymerase chain reaction (qPCR), next-generation sequencing (NGS), and fluorescence *in situ* hybridization (FISH), or (ii) evaluating treatment efficiency indirectly by using it to cure an artificially induced disease.

Distribution of specific species of microorganisms is still being studied in healthy humans and compared with those of patients with various gastrointestinal diseases. Perturbations of microbiota, even in case of alterations in numbers of a single species (i.e., *A. muciniphila*), might be a cause (and an indicator) of the development of disease (Karlsson et al., 2012). In this case, probiotic treatment might be useful in restoring microbiota balance in the gut. An interesting example of quantitative/qualitative analysis of animal gut microbiota after probiotic administration can be found in the study by Wang et al. (2015b): 454 pyrosequencing of fecal bacterial 16S rRNA genes in obese vs. lean mice showed that the probiotic strains shifted the overall structure of the gut microbiota of obese animals toward that of lean mice fed a normal diet, with significant changes observed in 83 operational taxonomic units. Due to complicated analyses required to understand specific mechanisms of disease development, as well as the mode of action of a certain probiotic microorganism, the use of disease models is generally more widespread.

### Rodent Models of GIT Diseases

Generally, in order to establish a disease model, mice are infected with the pathogen or irritant either one time or continuously (Pawlowski et al., 2010; Bhinder et al., 2013). Subsequently, animals are treated with probiotics with concomitant monitoring of the disease symptoms and evaluation of changes in the gut microflora. Following this approach, Verdú et al. (2008) infected mice with *H. pylori* for 4–6 months to investigate the effect of probiotic therapy on upper gastrointestinal dysfunction induced by chronic *H. pylori* infection. The authors reported that with probiotic treatment delayed gastric emptying in mice normalized significantly faster post-eradication, compared to control groups, where the dysfunction was observed during 2 months after pathogen administration was ceased. Mice and rats have also been used to evaluate the efficacy of probiotics for the treatment of *Salmonella* and *E. coli* O157:H7 infections (Asahara et al., 2001, 2004), inflammatory bowel disease (Shiba et al., 2003) and immune suppression (Lollo et al., 2012). Asahara et al. (2001) showed that intestinal growth and subsequent extra-intestinal translocation of orally-infected *Salmonella typhimurium* in mice were inhibited during administration of probiotic *B. breve*. Later, the same group reported *B. breve* was also effective in protecting mice against Shiga toxic-producing *E. coli* 0157:H7 (Asahara et al., 2004). Extrapolation of results achieved in animal studies and *in vitro* experiments to humans remains a difficult challenge. Many factors, such as differences in physiology and microflora composition of respective gastrointestinal systems, must be considered before interpreting the outcome.

The majority of *in vivo* experiments investigating the effects of probiotics on pathogenic bacterial populations use gnotobiotic mice (usually with human microflora systems in their GIT; Bernet-Camard et al., 1997; Aiba et al., 1998; Gill et al., 2001; Pawlowski et al., 2010). For instance, in a study by Shiba et al. (2003), probiotic *B. infantis* 1222 was found to significantly suppress the systemic antibody response raised by *Bacteroides vulgates*, a representative pathogenic *Bacteroides* sp. species, in a gnotobiotic mice model of inflammatory bowel disease. The use of conventional mice as a model for investigating human diseases is more problematic due to significant differences in animal and human gut microflora. Nevertheless, it is possible to use murine-specific organisms as models for the study of human pathogens. For instance, Ge et al. (2001) used *H. hepaticus* infection as an animal model for examining the pathogenesis of gastrointestinal diseases in humans caused by *H. pylori.* More recently, Bhinder et al. (2013) described the *Citrobacter rodentium* mouse model for the study of pathogen and host contributions during infectious colitis. *C. rodentium* is a murine-specific bacterial pathogen, closely related to enteropathogenic and enterohaemorrhagic strains of *E. coli* (Borenstein et al., 2008). Several *C. rodentium* infection studies involving mice models have shown probiotics to reduce the severity of symptoms and prevent death caused by the pathogenic agent (Chen et al., 2005; Gareau et al., 2010; Mackos et al., 2013). Chen et al. (2005) successfully treated *C. rodentium*-induced murine colitis with probiotic *L. acidophilus*. Gareau et al. (2010) similarly reported that *L. rhamnosus*, combined with *L. helveticus*, were effective in prevention and treatment of the same disease state in mice. Later, another group showed that *L. reuteri* was able to attenuate the severity of murine colitis caused by *C. rodentium* (Mackos et al., 2013). Further investigation of host–pathogen and probiotic–pathogen interactions will likely provide better insight into treatment of *C. rodentium* infection in mice, and possibly *E. coli* infections in humans. However, confirmation of probiotic benefits and possible side effects will ultimately require human trials.

### Human Clinical Trials

Human studies generally take the form of randomized clinical trials involving participants with some type of intestinal disorder. After assessment of eligibility and recruitment, participants are given either probiotic treatment or a placebo as a control. Results of these experiments have provided enough evidence for considering probiotics an efficient treatment for multiple GIT-associated diseases, such as acute gastroenteritis (Huang et al., 2002), irritable bowel syndrome (Nikfar et al., 2008) and necrotizing enterocolitis (Alfaleh and Anabrees, 2014). Some trials showing the efficacy of bacteria of interest in the treatment of specific gastrointestinal disorders are listed in **Table 3**. In one recent trial aimed to assess the efficiency of *S. cerevisiae* in treatment of irritable bowel syndrome, 179 adults, diagnosed with this condition, were randomized to receive once-daily 500 mg of *S. cerevisiae* or placebo for 8 weeks. Cardinal symptoms (abdominal pain/discomfort, bloating/distension, bowel movement difficulty) were recorded daily after a 2-week run-in period. The results showed that abdominal pain/discomfort scores were significantly reduced during probiotic intake (Pineton de Chamburn et al., 2015). A major trial involving 362 participants was conducted by Whorwell et al. (2006) in order to study the effect of *B. infantis* on symptoms of irritable bowel syndrome: probiotic administration lead to improvements in the majority of symptoms by more than 20%, compared to placebo. Another human clinical trial proved the efficacy of *Lactobacillus GG* in treatment of *H. pylori* infection: daily administration of the probiotic led to significant reduction in disease symptoms (diarrhea, nausea, and taste disturbances; Armuzzi et al., 2001). In general, data from multiple lines of research involving humans suggests that probiotic bacteria suppress gastrointestinal pathogens by simple competition by prevailing in numbers, and by producing antibacterial factors (bacteriocins and small organic molecules, such as fatty acids). Though more details into the mechanisms of action of probiotics on gut microbiota are essential, the large base of evidence already collected has proven the beneficial role in prevention and treatment of various GIT diseases in humans.

**Table 3:** Some of the major human trials of probiotics for the treatment of gastrointestinal diseases.

Probiotic strain	Disease	Number of participants	Reported outcome	Reference
*Lactobacillus rhamnosus* GG	*H. pylori* infection	60	Significant reduction (*p* = 0.04) of diarrhea, nausea and taste disturbances in the treatment group.	Armuzzi et al. (2001)
	Antibiotic-associated diarrhea in children	188	Significant reduction of the incidence of antibiotic-associated diarrhea in children treated with oral antibiotics for common childhood infections.	Vanderhoof et al. (1999)
		167	The treatment effect on the incidence of diarrhea (95% confidence interval) was -11% (-21-0%).	Arvola et al. (1999)
*B. bifidum*	Irritable bowel syndrome	122	Overall responder rates (decrease in symptoms severity) were 57% in the treatment group, but only 21% in the placebo group (*P* = 0.0001).	Guglielmetti et al. (2011)
*B. infantis*		362	The improvement in overall symptom assessment exceeded the placebo by more than 20% (*p* < 0.02).	Whorwell et al. (2006)
*S. cerevisiae*		179	The proportion of responders reporting improvement in abdominal pain/discomfort was significantly higher (*p* = 0.04) in the treated group than the placebo group (63% vs. 47%, OR = 1.88, 95%, CI: 0.99–3.57).	Pineton de Chamburn et al. (2015)
VSL#3^∗^	Pouchitis	40	Three patients (15%) in the treatment group had relapses of the disease within the 9-months follow-up period, compared with 20 (100%) in the placebo group (*P* < 0.001).	Gionchetti et al. (2000)
		40	Two of the 20 patients (10%) in the treatment group had an episode of acute pouchitis compared with 8 of the 20 patients (40%) treated with placebo (log-rank test, *z* = 2.273; *P* < 0.05).	Gionchetti et al., 2003
		34	Treatment of patients with mild to moderate stages of disease, not responding to conventional therapy, with probiotic resulted in a combined induction of remission/response rate of 77% with no adverse events.	Bibiloni et al. (2005)
	Ulcerative colitis	124	The efficacy of probiotic was significant (recurrence rate 34.6%, compared with 64.7% on placebo; *p* = 0.04) in patients with recurrent CDD, but not in patients with initial CDD (recurrence rate 19.3% compared with 24.2% on placebo; *p* = 0.86).	McFarland et al. (1994)
*Saccharomyces boulardii*	*Clostridium difficile-*associated diarrhea (CDD)	168	A significant decrease in recurrence of CDD was observed only in patients treated with high-dose vancomycin (2 g/day) and probiotic (16.7%) compared with those who received high-dose vancomycin and placebo (50%; *p* = 0.05).	Surawicz et al. (2000)
		211	The mean (+/-SD) duration of diarrhea was 1.69 days (0.6) in patients given probiotic, compared with 2.81 days (0.9) in those given placebo.	Buydens and Debeuckelaere (1996)
*Enterococcus faecium* SF68	Antibiotic-associated diarrhea	123	The probiotic was shown to be effective in reducing the incidence of antibiotic-associated diarrhea (AAD) in comparison with placebo (8.7% compared with 27.2%, respectively).	Wunderlich et al. (1989)
Mixture of lactobacilli, bifidobacteria and streprococci	Travelers’ diarrhea	94	Prophylaxis with the probiotic significantly decreased the frequency of diarrhea from 71 to 43% (*p* = 0.019).	Black et al. (1989)
Mixture of *B. infantis, B. bifidum, B. longum* and *L. acidophilus*	Necrotizing enterocolitis in newborns	186	Enteral administration of the probiotic in neonatal intensive care setup significantly reduced morbidity due to necrotising enterocolitis in very low birth weight newborn.	Samanta et al. (2009)

*^∗^A mixture of *Lactobacillus casei, L. plantarum, L. acidophilus, L. delbrueckii subsp. bulgaricus, B. longum, B. breve, B. infantis*, and *Streptococcus salivarius* sp. *Thermophiles*.*

## Conclusion

Many strains of genera *Lactobacillus* and *Bifidobacterium*, as well as some enterococci and yeasts, have been shown to possess probiotic properties with potential for prophylaxis and treatment of a range of gastrointestinal disorders. The effectiveness of probiotic bacteria in the treatment of these conditions is supported by many clinical trials involving patients of all ages and probiotic organisms chosen based on laboratory research trials. Notably, most of the work in the probiotic field has been conducted *in vitro*, as it is an essential step in the investigation of bacterial growth, metabolite production, ability to form biofilms, compete with pathogens, co-aggregate, and produce antimicrobials. All of these characteristics are important factors for identification of potential probiotic strains that possess desirable properties along with the ability to establish itself in the human gut. Intrinsic antibiotic resistance and transferability of genetic determinants are two additional factors to account for at the initial stage of a probiotic study. Novel putative probiotic species, such as *A. muciniphila*, are yet to be tested in both animal and human trials; however, the results achieved to date suggest that they might be beneficial in treatment or diagnosis of GIT diseases.

## Conflict of Interest Statement

The authors declare that the research was conducted in the absence of any commercial or financial relationships that could be construed as a potential conflict of interest.
